# Association of an intact *E2* gene with higher HPV viral load, higher viral oncogene expression, and improved clinical outcome in HPV16 positive head and neck squamous cell carcinoma

**DOI:** 10.1371/journal.pone.0191581

**Published:** 2018-02-16

**Authors:** Nicole V. Anayannis, Nicolas F. Schlecht, Miriam Ben-Dayan, Richard V. Smith, Thomas J. Belbin, Thomas J. Ow, Duk M. Blakaj, Robert D. Burk, Sarah M. Leonard, Ciaran B. Woodman, Joanna L. Parish, Michael B. Prystowsky

**Affiliations:** 1 Department of Pathology, Albert Einstein College of Medicine and Montefiore Medical Center, Bronx, United States of America; 2 Department of Epidemiology & Population Health, Albert Einstein College of Medicine and Montefiore Medical Center, Bronx, United States of America; 3 Department of Medicine (Oncology), Albert Einstein College of Medicine and Montefiore Medical Center, Bronx, United States of America; 4 Department of Cancer Prevention and Control, Roswell Park Cancer Institute, Buffalo, NY, United States of America; 5 Department of Otorhinolaryngology-Head and Neck Surgery, Albert Einstein College of Medicine and Montefiore Medical Center, Bronx, United States of America; 6 Discipline of Oncology, Faculty of Medicine, Memorial University of Newfoundland, St. John’s, Newfoundland, Canada; 7 The James Cancer Center, Ohio State University, Columbus, OH, United States of America; 8 Department of Pediatrics (Genetics), Albert Einstein College of Medicine and Montefiore Medical Center, Bronx, United States of America; 9 Department of Microbiology & Immunology, Albert Einstein College of Medicine and Montefiore Medical Center, Bronx, United States of America; 10 Department of Obstetrics, Gynecology & Women’s Health, Albert Einstein College of Medicine and Montefiore Medical Center, Bronx, United States of America; 11 Institute of Cancer and Genomic Sciences, College of Medical and Dental Sciences, University of Birmingham, Edgbaston, Birmingham, United Kingdom; Fondazione IRCCS Istituto Nazionale dei Tumori, ITALY

## Abstract

To assess the relationship of *E2* gene disruption with viral gene expression and clinical outcome in human papillomavirus (HPV) positive head and neck squamous cell carcinoma, we evaluated 31 oropharyngeal and 17 non-oropharyngeal HPV16 positive carcinomas using two PCR-based methods to test for disruption of *E2*, followed by Sanger sequencing. Expression of HPV16 *E6*, *E7* and *E2* transcripts, along with cellular *ARF* and *INK4A*, were also assessed by RT-qPCR. Associations between *E2* disruption, E2/E6/E7 expression, and clinical outcome were evaluated by Kaplan-Meier analysis for loco-regional recurrence and disease-specific survival. The majority (n = 21, 68%) of HPV16 positive oropharyngeal carcinomas had an intact *E2* gene, whereas the majority of HPV16 positive non-oropharyngeal carcinomas (n = 10, 59%) had a disrupted *E2* gene. Three of the oropharyngeal tumors and two of the non-oropharyngeal tumors had deletions within *E2*. Detection of an intact *E2* gene was associated with a higher DNA viral load and increased *E2/E6/E7*, *ARF* and *INK4A* expression in oropharyngeal tumors. Oropharyngeal carcinomas with an intact *E2* had a lower risk of loco-regional recurrence (log-rank p = 0.04) and improved disease-specific survival (p = 0.03) compared to tumors with disrupted *E2*. In addition, high E7 expression was associated with lower risk of loco-regional recurrence (p = 0.004) as was high E6 expression (p = 0.006). In summary, an intact *E2* gene is more common in HPV16 positive oropharyngeal than non-oropharyngeal carcinomas; the presence of an intact *E2* gene is associated with higher HPV viral load, higher viral oncogene expression, and improved clinical outcome compared to patients with a disrupted *E2* gene in oropharyngeal cancer.

## Introduction

Each year head and neck squamous cell carcinoma (HNSCC) accounts for 550,000 cancer cases worldwide, resulting in 300,000 deaths [1]. A subset of HNSCC is associated with human papillomavirus (HPV) infection, with most arising in the oropharynx. More than 150 alpha HPV types have been identified [2] of which a subset is considered oncogenic [3] in several human cancers including cervical, penile, vulvovaginal and anal carcinomas (Munger, 2004). HPV16 is one such oncogenic, or high-risk type, found in over 90% of HPV positive oropharyngeal squamous cell carcinomas (OPSCCs) [4]. HPV16 positive OPSCCs have been shown to have a better prognosis than HPV16 negative OPSCCs [5,6].

The HPV16 virus is a double-stranded circular DNA virus that is 8 kb in length, and encodes six early (*E*) genes, *E1*, *E2*, *E4*, *E5*, *E6*, *E7* and two late (*L*) genes: *L1* and *L2 [7]*. The L1 and L2 proteins form the viral capsid structure [8] and primers amplifying conserved regions of *L1* are used to test clinical samples for the presence of HPV DNA [9]. Two of the early gene products, E6 and E7, are oncogenes that disrupt tumor suppressor pathways and are consistently expressed following HPV16 infection [10]. E6 promotes ubiquitination and degradation of the tumor suppressor p53, impairing the cellular response to DNA damage. E7 binds to and inactivates the retinoblastoma protein (pRb), which controls the G1-S phase entry into the cell cycle [7]. Inhibition of pRb activity by E7 is in turn associated with overexpression of the cell cycle regulator p16, and p16 immunohistochemistry has been used as a surrogate test for HPV infection in OPSCC [5,6,11–13]. The p16 transcript, p16^INK4A^, is transcribed from the *CDKN2A* locus, which also encodes an alternative transcript p14^ARF^. Both p16^INK4A^ and p14^ARF^ are tumor suppressors [14] and over expression of both transcripts has been associated with HPV status in OPSCC [15].

The HPV16 genome can exist in the cell as an episome, or it can integrate into the human genome [16]. HPV episomes can be detected in non-malignant and pre-malignant tissues, while integrated HPV is detected largely in malignancies, particular in the cervix [17]. Disruption of the *E2* gene occurs frequently upon integration of the virus into the human genome [18], and has been correlated with increased expression of E6 and E7 *in vitro* [19,20]. However, correlations with HPV16 *E2* disruption and increased E6 and E7 expression in cervical cancer have been inconsistent [21–24]. Although the status of *E2* disruption in HNSCC was previously unknown, recent studies have analyzed integration of HPV in HNSCC, some with conflicting results [25]. A study on HNSCC cell lines did not find a correlation between HPV integration and viral expression, including *E2* mRNA levels [26].Another recent study of HPV positive head and neck cancer cell lines found that there was viral integration in all cell lines and that E6 and E7 transcripts were expressed in all of these cell lines [27]. However, a study of OPSCC tumor samples found HPV16 integration in only 2 of 13 tumors, with expression of E6, E7 and E2 being concordant [28]. Alternatively a recent analysis of HPV16 positive HNSCC data from the TCGA by Nulton et al. found three categories of HPV genomic DNA including episomal, integrated and human-viral episomal hybrids; three quarters of samples retained viral episomes or human-viral hybrid episomes which replicated by an E1-E2 dependent manner [29].

While disruption of the *E2* gene has been associated with poor prognosis in cervical cancer [30,31] this has been studied to a lesser extent in HNSCC [32,33]. In this study, we evaluated the status of the *E2* gene by directly testing for *E2* disruption in HPV16 positive OPSCC and non-OP HNSCC tumors to assess the relationship between *E2* disruption to viral oncogene expression, host gene expression and clinical outcome.

## Materials and methods

### Patients and samples

Patients with primary HNSCC at Montefiore Medical Center (MMC) in the Bronx, NY were enrolled in an ongoing study approved by the Institutional Review Boards of MMC and Albert Einstein College of Medicine. All patients who enrolled provided written, informed consent. Tumor samples were collected prior to therapy and snap frozen in liquid nitrogen within 30 minutes of biopsy or surgical resection. 31 OPSCC samples were selected and 17 non-OP HNSCC samples were selected. Samples selected for this study were restricted to HPV16 positive samples only, and were identified as HPV positive prior to this study by the methods described under HPV DNA detection.

### Cell culture

Two cervical cancer cell lines, CaSki and SiHa (American Type Culture Collection), and two oral cavity SCC cell lines, UPCI:SCC090 and UMSCC-47 (courtesy of Dr. Thomas Carey, University of MI)(48), were used as positive controls in this study. Cells were cultured in 10cm TC plates (Corning, Corning NY) and incubated at 37°C with 5% CO2. Caski cells were cultured in RPMI 1640 (Invitrogen, Grand Island NY) and SiHa cells were cultured in MEM with Earle's salts (Fisher, Hampton NH) with 0.01% 100mM (100X) sodium pyruvate (Gibco, Grand Island NY) and 0.01% non-essential amino acids (Hyclone). Cervical cancer cell lines were cultured in 10% FBS (Gibco) and 0.01% penicillin/streptomycin. Medium was changed every 2–3 days. UPCI: SCC090 cells were cultured in MEM with Earle's salts and UMSCC-47 cells were cultured in high glucose DMEM (Gibco). Cell lines were cultured in 0.01% L-glutamine (Hyclone, Logan UT), 0.01% penicillin/streptomycin, 0.01% non- essential amino acids and 10% FBS.

### RNA and DNA extraction

A standard TRIzol (Invitrogen, Carslbad, CA) protocol was used to extract total RNA. Tissue specimens were subdivided into two pieces. One piece was kept for RNA extraction and one piece for DNA extraction. For RNA extraction, tissue was homogenized in 1ml of TRIzol solution and cell lines were scraped from culture dishes into 1ml of TRIzol. DNA quality and quantity was assessed spectrophotometrically using a NanoDrop ND-1000 spectrophotometer (NanoDrop, Wilmington, DE). RNA quality was assayed on an Agilent Bioanalyzer and all RNA samples were stored at -80 C in ethanol [34]. DNA extraction from tissue specimens was done using the Qiagen protocol and reagents from the DNeasy Blood and Tissue extraction kit and handbook.

### DNase treatment

To ensure there was no DNA contaminant in RNA samples, all RNA used for experiments was DNase treated. A standard DNase kit (Promega, Madison WI) and protocol were used to ensure the RNA samples used for experiments did not contain DNA contaminants.

### DNA extraction from formalin-fixed and paraffin-embedded (FFPE) specimens

In order to confirm HPV16 status in fresh frozen samples, sections were taken from the formalin-fixed paraffin embedded blocks from the Pathology Department of Montefiore Medical Center. DNA was extracted from the FFPE blocks according to a Trizol-based method and ethanol precipitation described in Kotorashvili *et al*. 2012 [35]. After ethanol precipitation, one 24 hour incubation with 20ul of proteinase K was done followed by a second 48 hour incubation with 20ul of proteinase K. Afterwards samples were spun for one minute and FFPE-DNA was recovered from the lower phase of TRIzol using a Qiagen DNA FFPE kit (Valencia, CA) under standard protocol [36].

### HPV DNA detection and viral load evaluation

HPV DNA status was determined by PCR amplification using MY09/MY11/HMB01-PCR system with Gold AmpliTaq that amplifies a conserved 450 base-pair segment in the L1-sequence of HPV under standard protocols [37]. In order to determine type-specific HPV infections, PCR reactions using type- specific biotin-labeled probes were done and products were detected by dot blot hybridization [9]. The signal intensity was evaluated as a qualitative measure of viral load, the scale being 1–5, where 1 is weakest and 5 is highest. The score is based on density and diameter of the PCR product on the autoradiogram [38,39]. For this study we analyzed the PCR signal strength index scores 1–5 separately, in order to compare samples with an intact E2 gene and samples with a disrupted E2 gene. We first analyzed all HNSCC samples together. Then, we analyzed OPSCC as one group and non-OP tumors as a separate group. All analyses on viral load were done by Mann-Whitney test. Samples were tested independently for DNA and RNA. If results were discordant, samples were tested by additional PCR. Discordant specimens were omitted from the study if HPV16 status was unable to be confirmed through additional PCR.

### PCR detection of HPV16 *E2* and *E6* genes

**Fig 1:**
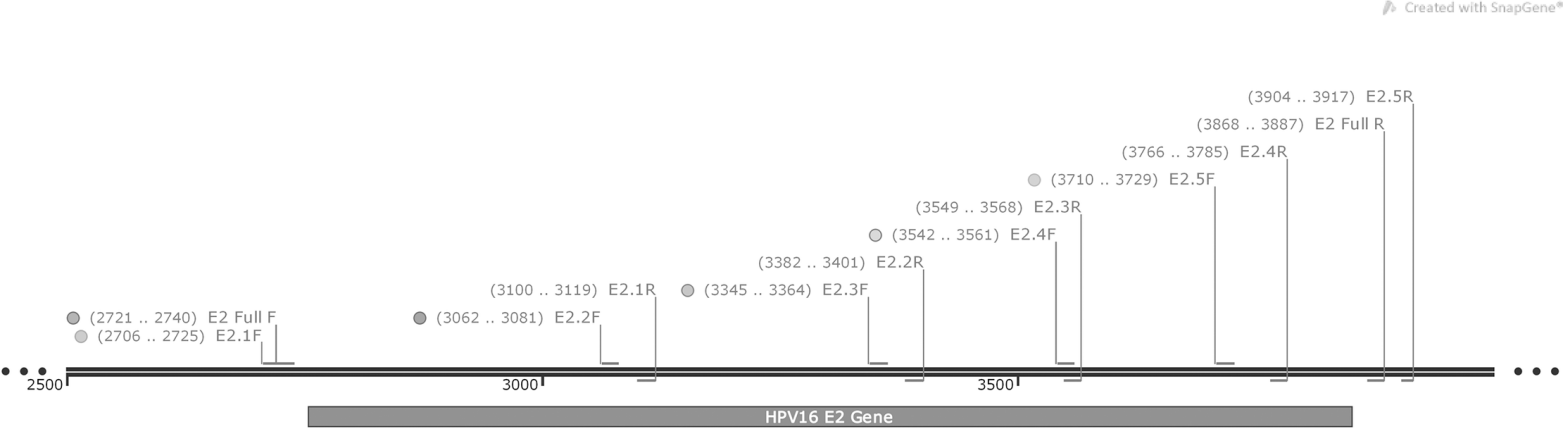
Positions of HPV16 E2 primers along the HPV16 E2 gene. The HPV16 genome encompassing the *E2* gene region shown in gray. Primer locations of the *E2* disruption assay and the *E2* full gene primer set are shown above and numbered according to nucleotides on the whole genome. Overlapping primer design is illustrated below along with product size primer set.

We used a previously published assay to determine the integrity of the *E2* gene [40]. This included five primer sets which sequentially span the length of the gene and a single primer set that detects the full length *E2* gene [22,40,41]. Primers for HPV16 *E6* DNA were also used as an additional HPV16 control. Primer locations are shown in **Fig 1** (figure designed with SnapGene software) and sequences as well as locations are given in **S1 Table**. DNA extracted from SiHa cells served as the *E2* disrupted control. SiHa has one integrated HPV16 genome which is disrupted at nucleotides 3132 and 3384, therefore the second primer set of the five primer set for the *E2* gene does not give a product for SiHa [10,40]. DNA from UPCI:SCC090 cells, which contain high copy numbers of HPV16 and was confirmed to harbor episomal virus, served as the *E2* intact control [42,43].

In order to validate the results of the initial *E2* disruption assays, we ran a PCR screen of all samples using primers designed to amplify the entire *E2* gene. A full length PCR product confirmed *E2* disruption assay results indicating presence of an intact *E2* gene for all cases identified by the five primer assay. Samples generating no band indicated presence of only disrupted *E2* gene products. A smaller product suggested a deletion. The presence of an intact *E2* gene or deletion was further confirmed by Sanger sequencing. Examples of the gene products are presented in **S1 Fig**.

PCR conditions were optimized in a previous study and carried out under the same conditions for all *E2* primers and E6 primers [40]. Briefly they were done with 12.5μl of 2X master mix (Thermoscientific, Waltham MA), 3μl of primer (2.5 pmol forward + reverse) and 20ng of DNA, brought up to a total reaction volume of 25μl. Cycling conditions were: 95°C for 5 min, followed by 60 cycles of 95°C for 30s, melting temperature of 55°C for 1 min and 72°C for 2 min, and a final extension of 72°C for 10 min. Products from the five primer sets of the *E2* disruption assay were then electrophoresed on a 2% agarose gel. Products from the full length *E2* PCR reaction were analyzed on a 1% agarose gel [40].

### Quantitative Real-Time PCR

The DNase treated RNA and untreated RNA was diluted to 100ng/μl for reverse transcription. Expression of HPV16 genes in tissue samples was measured using TaqMan® RNA-to-CT™ 1-Step RT-PCR Master Mix Kits (Applied Biosystems, Carlsbad CA) with probes for E6, E7, E2-3’ & E2-5’ (primer and probe sequences are given in **S2 Table**). All gene expression was normalized to the control probe glyceraldehyde-3-phosphate dehydrogenase. Assays were performed in triplicate on the StepOnePlus™ Real-Time PCR apparatus (Applied Biosystems) [34,44].

### Statistical analysis

We first tested for statistical differences in the proportions of HPV16 positive OPSCC and non-OPSCC tumors that had any intact vs. only disrupted E2 gene using the Fisher’s exact test. Box-and-Whisker plots and Mann-Whitney U tests were used to determine the statistical significance of observed differences in transcript levels for viral E6, E7 and E2 oncogenes, for *CDKN2A* INK4A and ARF, and for HPV16 DNA viral load, comparing OPSCC and non-OPSCC tumors with any intact vs. only disrupted E2. Transcript levels generated by RT-qPCR were estimated using 2maxΔCT- ΔCT, where maxΔCT was the lower limit threshold of detection, and ΔCT was the experimental value minus the endogenous control (GAPDH). HPV16 DNA viral load was derived from dot blot hybridization intensity scores. Statistical analyses were done separately for OPSCC and non-OPSCC tumor groups using GraphPad Prism software (San Diego, CA), and all tests were two-sided.

We used contingency tables to assess the associations between E2 status (i.e., comparing tumors any intact vs. only disrupted E2 gene) and clinicopathologic factors at diagnosis (e.g., age, gender, tumor stage), and tested for statistical differences using Fisher Exact tests. Associations between E2 status and clinical outcome (for local regional recurrence and cancer death) were assessed for OPSCC tumors only, using Kaplan-Meier plots and Log-rank (Mantel- Cox) tests. Associations with clinical outcome for OPSCC tumors were also assessed for HPV16 E7 or E6 expression grouping tumors into low vs. high expression based on median cut-point using derived (2maxΔCT- ΔCT) RT-qPCR results.

## Results

### The HPV16 *E2* gene is intact in OPSCC and disrupted in non-OP HNSCC tumors

We tested a total of 31 oropharyngeal and 17 non-oropharyngeal HPV16 positive tumors for disruption of the HPV16 *E2* gene by PCR analysis and found that of the 31 HPV16 positive OPSCCs tested, 21 (68%) contained intact or possibly mixed (both intact and disrupted) E2 gene products and 7 (22%) contained only disrupted *E2*. Five (29%) of the 17 HPV16 positive non-OPSCC tumors were found to have an intact *E2*, while 10 (59%) had a disrupted *E2* (**Table 1**). In addition, there were five tumors with deletions in the *E2* gene (3 OP and 2 non-OP), which are described in the following section. There was a significant difference between the oropharyngeal and non-oropharyngeal tumors with respect to the presence of intact *E2* or disrupted *E2* gene products (Fisher’s exact test, p = 0.01) (**Table 1**).

**Table 1:** Study population characteristics stratified by HPV16 *E2* gene status and tumor site.

**Oropharynx**^**3**^**, n = 31**							
	Intact E2		Disrupted E2		p value*	Deletions	
	n = 21 (68%)		n = 7 (22%)		0.01^1^	n = 3 (10%)	
**Age at diagnosis in years**							
**<60**	12	57%	3	43%	0.4	0	0%
**≥60**	9	43%	4	57%		3	100%
**Sex**							
**Men**	18	86%	6	86%	1	1	33%
**Women**	3	14%	1	14%		2	67%
**Smoking Status**^**2**^							
**Current Smoker**	5	24%	3	43%	0.42	1	33%
**Ex-smoker**	12	57%	2	29%		1	33%
**Never Smoker**	4	19%	2	29%		1	33%
**Overall Stage**							
**I–II**	3	14%	1	14%	1	1	33%
**III–IV**	18	86%	6	86%		2	67%
**Nodal Stage n (%)**							
**N0**	3	14%	1	14%	0.8	1	33%
**N1**	1	5%	0	0%		0	0%
**N2**	14	67%	4	57%		2	67%
**N3**	3	14%	2	29%		0	0%
**Tumor Size n (%)**							
**T1–T2**	17	81%	4	57%	0.32	2	67%
**T3–T4**	4	19%	3	43%		1	33%
**Anatomical Site**^**3**^**, n (%)**							
**Tonsil**	14	67%	2	29%	0.05	2	67%
**Base of Tongue**	6	28%	2	29%		1	33%
**Oropharyngeal Wall**	0	0%	2	29%			
**Not Specified**	1	5%	1	14%			
**Non-oropharynx, n = 17**							
	Intact E2		Disrupted E2		p value*	Deletions	
**Age at diagnosis in years**	n = 5 (29%)		n = 10 (59%)		0.01^1^	n = 2 (12%)	
**<60**	3	60%	6	60%	0.61	1	50%
**≥60**	2	40%	4	40%		1	50%
**Sex, n (%)**							
**Men**	4	80%	7	70%	1	2	100%
**Women**	1	20%	3	30%		0	0%
**Smoking Status**^**2**^							
**Current Smoker**	1	20%	5	50%	0.47	0	0%
**Ex-smoker**	3	60%	3	30%		2	100%
**Never Smoker**	1	20%	2	20%		0	0%
**Overall Stage**							
**I–II**	1	20%	2	20%	1	0	0%
**III–IV**	4	80%	8	80%		2	100%
**Nodal Stage n (%)**							
**N0**	2	40%	4	40%	0.49	0	0%
**N1**	1	20%	2	20%		0	0%
**N2**	1	20%	4	40%		2	100%
**N3**	1	20%	0	0%		0	0%
**Tumor Size n (%)**							
**T1–T2**	3	60%	4	40%	0.61	0	0%
**T3–T4**	2	40%	6	60%		2	100%
**Anatomical Site**^**3**^**, n (%)**							
**Oral Cavity**	1	20%	4	40%	74	0	0%
**Larynx**	2	40%	3	30%		1	50%
**Hypopharynx**	2	40%	3	30%		0	0%
**Nasopharynx**	0	0%	0	0%		1	50%

*p-Value for 2-sided Fisher exact test. Row numbers may not sum to column totals due to missing data.

1p-value determined by Fisher exact or Chi-square test on results from intact and disrupted *E2* status for oropharynx and non-oropharynx.

2Smoking status was defined as never smoked, exsmoker (at time of diagnosis) and current smoker

^3^Oropharynx includes: base of tongue, tonsil, soft palate, oropharyngeal wall, uvula and oropharynx-NOS. Hypopharynx includes: posterior pharyngeal wall, pyriform sinus and hypopharynx-NOS. Larynx includes glottis, supraglottis-aryepiglottic fold and epiglottis. Oral cavity includes buccal, alveolar ridge, anterior tongue, floor of mouth, hard palate, inner lip mucosa and retromolar trigone.

As noted in Table 1, five samples demonstrated a deletion in the E2 gene. Deletions in all three of the OPSCC samples were found in the hinge region between nucleotides 3394–3474, 3573–3596 and 3577–3594. Deletions found in the two non-OP tumor samples occurred in the transactivation domain and hinge regions (nucleotides 3293–3306 and 2766–2816, respectively). We ran the E2 sequences from the OPSCC samples with deletions through Geneious 10.2.2 and based on the sequence, these are complete ORFs that can produce transcripts and do not result in a frame shift.

### The HPV16 E6, E7 and E2 are expressed at higher levels in OPSCCs with an intact *E2* gene

**Fig 2:**
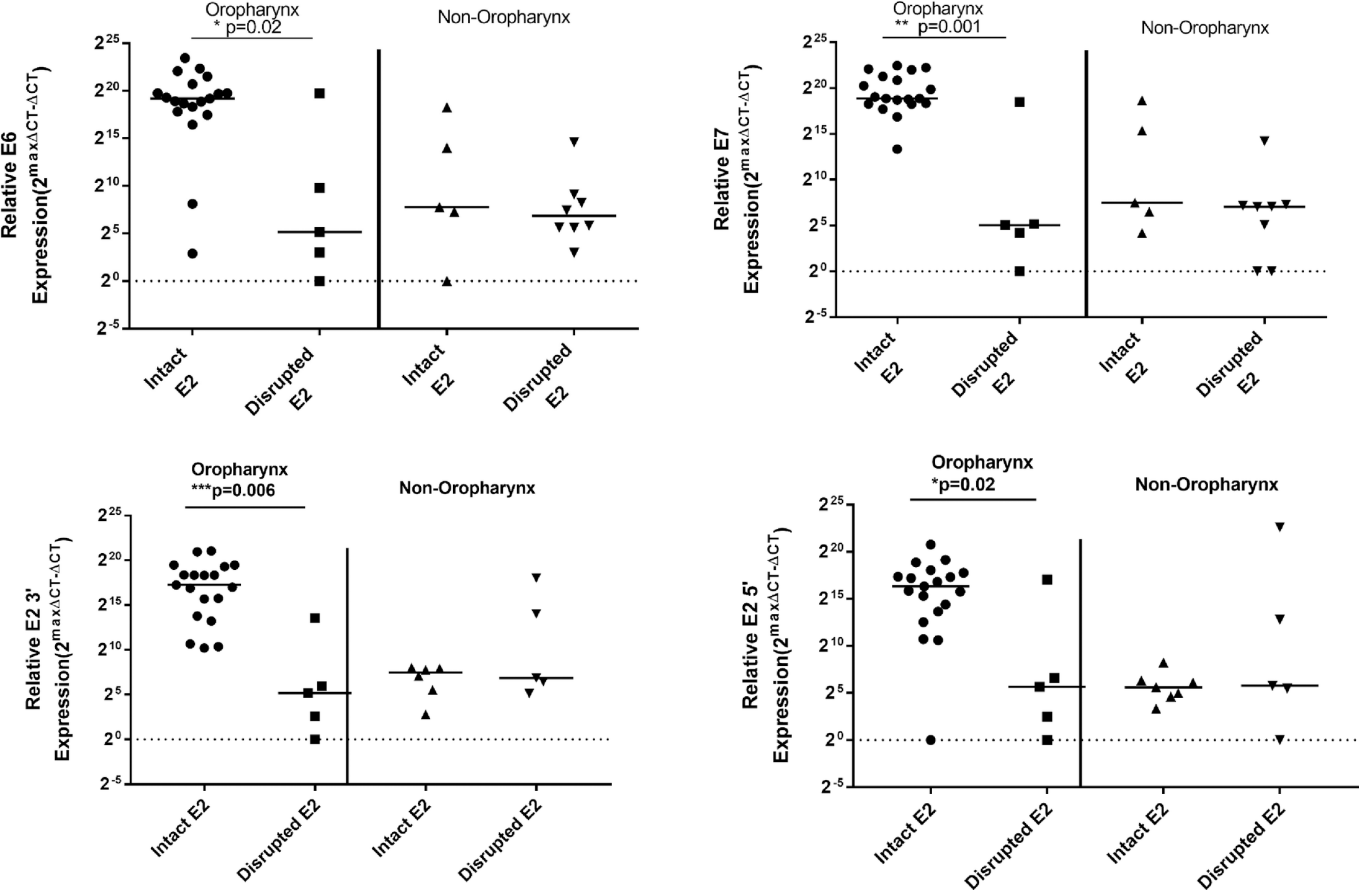
HPV16 E6, E7 and E2 are overexpressed in OPSCCs with an intact *E2*. HPV16 E6 (A) and HPV16 E7 (B) expression with HPV16 *E2* gene disruption. Expression is defined as Y = 2^maxΔCT- ΔCT^ where maxΔCT is the lower limit threshold of detection which equals 20 or 1. ΔCT is the experimental value minus the endogenous control (GAPDH). Statistical significance for difference in HPVE6 and E7 expression by site and between samples with an intact or disrupted HPV16 *E2* gene was assessed by Mann-Whitney test.

We next determined the prevalence of *E2* disruption in HPV16 positive HNSCC and the correlation between *E2* disruption and E6/E7 viral oncogene expression in our HPV16 positive HNSCC samples (**Fig 2**). We evaluated RNA levels for both oncogenes in 19 of the intact and 5 of the disrupted OPSCCs. Of the non-OP tumors we evaluated viral expression in 5 of the tumors with an intact *E2* gene and 8 of the tumors with a disrupted *E2* gene. Due to limited availability of RNA from clinical samples, the full sample set could not be tested. We found that *E2* disruption was significantly associated with lower expression of both E6 (2A; Mann-Whitney test p = 0.02) and E7 (2B; p = 0.001) in OPSCCs. An association between intact *E2* and E6 / E7 expression was not observed in the non-OPSCC samples.

In the 28 oropharyngeal tumors, those cases with an intact *E2* gene had significantly higher E6 and E7 expression than the oropharyngeal cases with a disrupted *E2* gene (Mann Whitney test, p = 0.02 and p = 0.001). HPV16 E2 3’ (C) and 5’ (D) expression is also significantly higher in oropharyngeal cancers cases with an intact *E2* gene than the oropharyngeal cases with a disrupted *E2* gene (Mann Whitney test, p = 0.0006 and p = 0.02). There was no significant difference in expression of E2 in non-oropharyngeal HNSCC tumors. With the E2 protein containing a DNA-binding domain, located at the 3’ end of the *E2* gene, and a transactivation domain at the 5’ end, we designed primers specific to the 3’ and 5’ regions of the E2 transcript to test for expression of the full length transcript (**Fig 2C and 2D**). RNA levels of the E2 transcript corresponding to DNA-binding and transactivation domain were high in OPSCC samples with an intact *E2* gene compared to those with disrupted *E2* (Mann-Whitney test p = 0.0006 for E2 3’and p = 0.02 for E2 5’), indicating that the full length transcript is present in the cell. No association was observed between *E2* disruption and 3’ or 5’ domain transcript levels in non-OPSCCs.

### HPV16 viral load is higher in HNSCCs with an intact *E2* gene regardless of anatomic site

**Fig 3:**
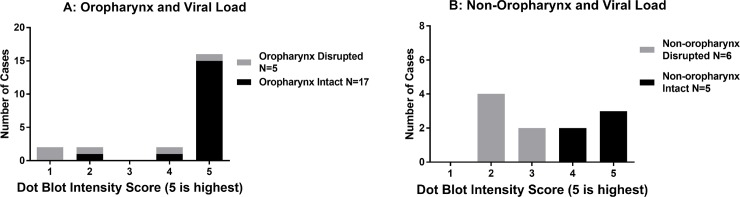
Low HPV16 DNA viral load correlates with *E2* gene disruption in HPV16 positive OPSCC (A) and non-OP tumors (B). Viral load was determined by dot-blot intensity and scored from 1–5, 5 being highest. Scores are graphed separately across the x-axis. Samples with an intact HPV16 *E2* gene are represented by the grey bars and samples with a disrupted HPV16 *E2* gene are represented by the black bars.

Since our findings that expression of E6 and E7 was high in OPSCCs with an intact *E2* gene, which is counter to findings of studies in cervical cancer, we hypothesized that viral load might explain in part the increased oncogene expression observed in tumors with an intact *E2* gene (**Fig 3**). Tumors with intact *E2* genes were significantly more likely to have higher viral loads than those with disrupted *E2* for both OPSCC (3A; Mann Whitney test p = 0.002) and non-OPSCC (4B; p = 0.003) patients.

**Fig 4:**
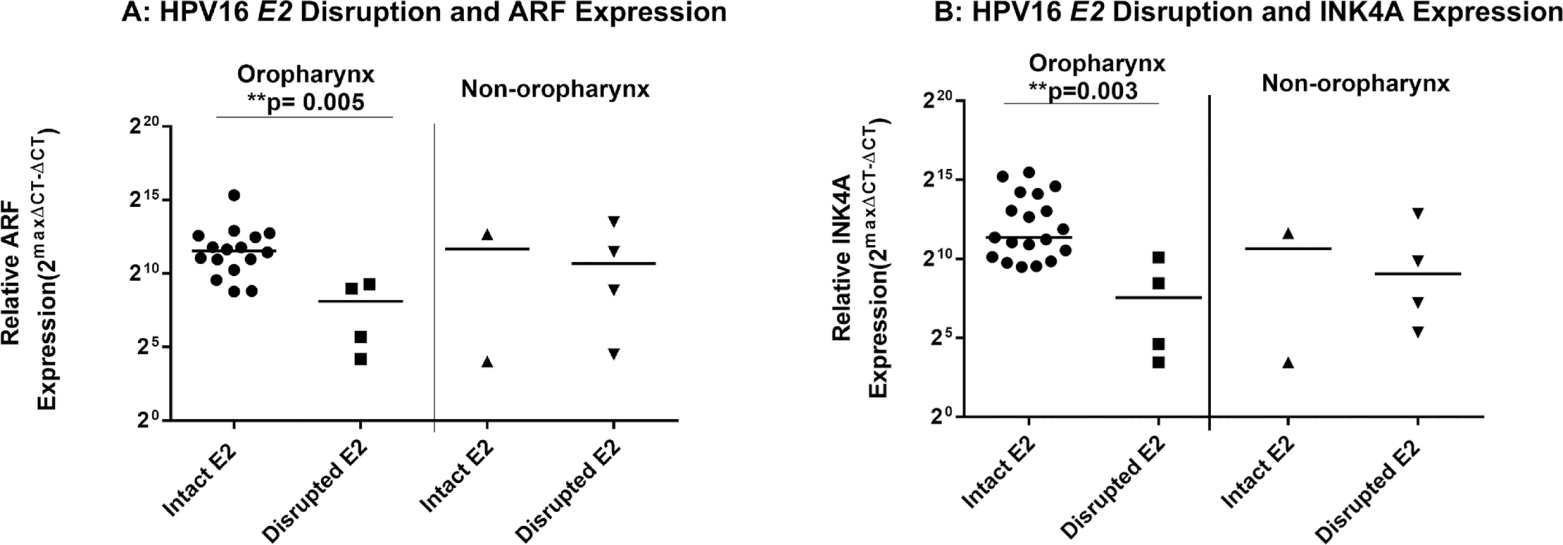
ARF and INK4A are overexpressed in OPSCCs with an intact E2. ARF (A) and INK4A (B) expression with HPV16 *E2* gene disruption. Expression is defined as Y = 2maxΔCT- ΔCT where maxΔCT is the lower limit threshold of detection which equals 20 or 1. ΔCT is the experimental value minus the endogenous control (GAPDH). Statistical significance for difference in HPVE6 and E7 expression by site and between samples with an intact or disrupted HPV16 *E2* gene was assessed by Mann-Whitney test.

Tumors with intact *E2* genes were significantly more likely to have higher viral loads than those with disrupted *E2* for both the oropharynx (3A) (p = 0.002) and non-oropharynx (3B) (p = 0.003). Tumor suppressor p14^ARF^ and p16^INK4A^ expression is higher in OPSCCs with an intact *E2* gene. Our previous research revealed that HPV16 positive OPSCC have higher expression of CDKN2A locus transcripts than HPV16 negative OPSCC. In order to determine if CDKN2A expression also correlated with E2 gene status, we measured p14^ARF^ and p16^INK4A^ RNA levels in our tumor samples (**Fig 4A and 4B**). Sixteen OPSCC samples with an intact *E2* gene were tested for ARF expression and 17 samples were tested for p16^INK4A^. Four disrupted OPSCCs were tested for both. Two *E2* intact and 4 disrupted non-OP tumors were tested. Sample size for these experiments was limited due to availability of RNA. Similar to our findings with E6 and E7 expression, p14^ARF^ and p16^INK4A^ were expressed at significantly higher levels in OPSCC with intact vs. disrupted E2 gene products (Mann-Whitney test p = 0.005 and p = 0.004, respectively). There were no significant differences observed with p14^ARF^ or p16^INK4A^ expression in non-OPSCC.

Similar to our results for E6 and E7 expression, in oropharyngeal tumors, those cases with an intact E2 gene had significantly higher ARF and INK4A expression then the oropharyngeal cases with a disrupted *E2* gene (Mann Whitney test, p = 0.005 and p = 0.004). There was no significant difference in expression of ARF and INK4A between non-oropharyngeal HNSCC tumors with an intact and disrupted E2.

### Disruption of the *E2* gene and expression of the E7 oncogene are associated with local-regional recurrence and disease-specific survival

**Fig 5:**
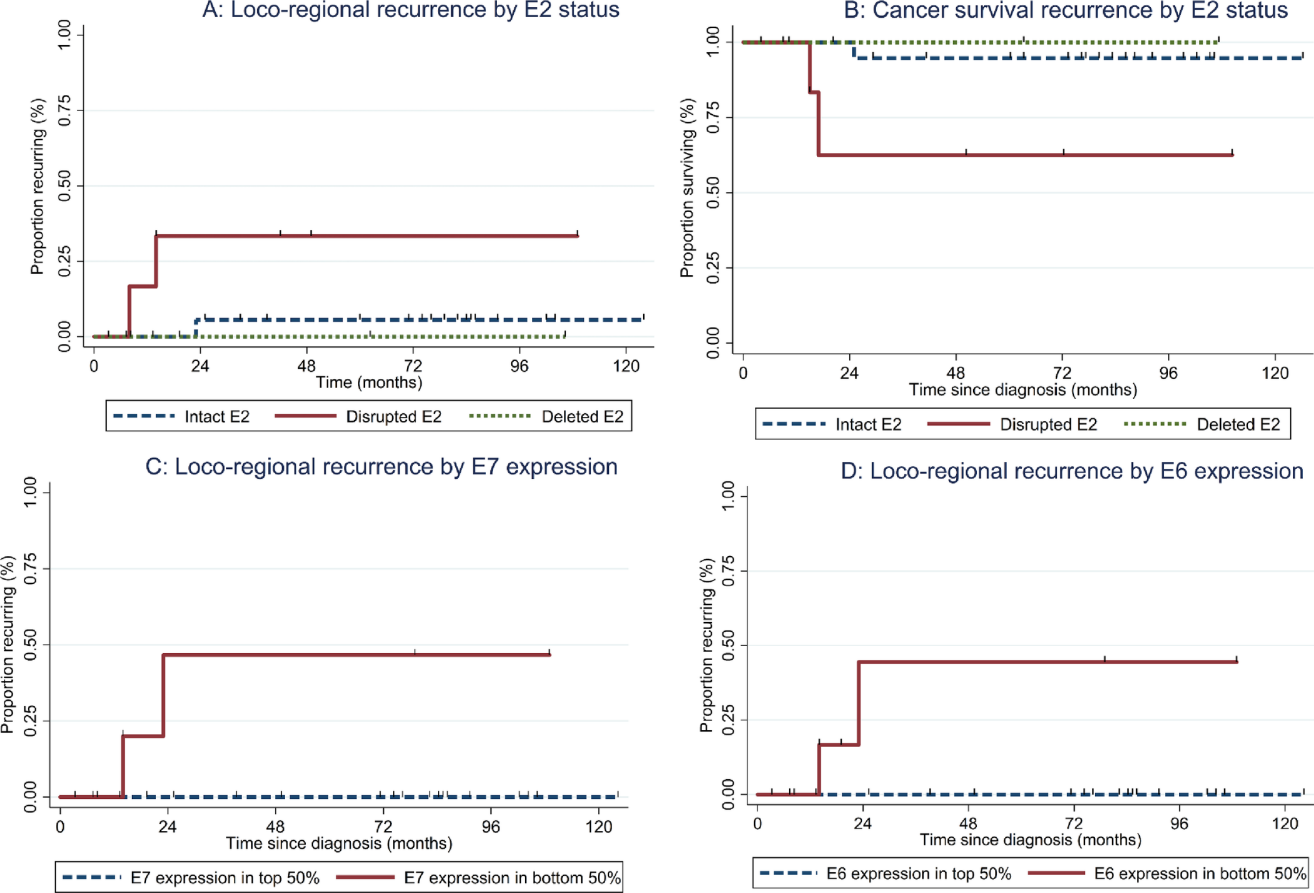
Association of local-regional recurrence and disease specific survival with HPV16 *E2* disruption in the oropharynx. (A) Samples with an intact *E2* gene (broken black line) have a lower incidence of local regional recurrence than oropharyngeal cases with a disrupted *E2* gene (solid black line) (Mantel-Cox test p = 0.04). (B) Samples with an intact *E2* gene (broken black line) have better disease specific survival than oropharyngeal cases with a disrupted *E2* gene (solid black line) (Mantel-Cox test p = 0.03). (C and D) Samples were separated into high (broken black) or low (solid black) expression of viral genes based on median cutoff using derived (2maxΔCT- ΔCT) qRT-PCR results compared to GAPDH for: (C) HPV16 E7 (median = 1.69 2maxΔCT- ΔCT; Mantel-Cox test p = 0.004), and (D) HPV16 E6 (median = 1.23 2maxΔCT- ΔCT; Mantel-Cox test p = 0.006).

Studies in both cervical and oropharyngeal cancers have reported *E2* disruption to be associated with poor prognosis. We further explored the relationship between *E2* disruption, oncogene expression and clinical outcome in our cohort of HPV16 positive OPSCC patients. Our clinical analysis was done on data collected from patients followed prospectively after diagnosis and treatment. Patients are followed until time of death or withdrawal from the study. We found that tumors derived from the oropharynx containing only a disrupted *E2* gene had higher risk of local-regional recurrence (**Fig 5A**; Mantel-Cox test, p = 0.04) and poorer disease-specific survival (**Fig 5B**;p = 0.03) compared to HPV16 positive OPSCC tumors with an intact *E2* gene status.

Based on our data showing that the presence of an intact *E2* gene was associated with high E6 and E7 expression in OPSCC, and that HPV16 positive OPSCC with disrupted *E2* also had poorer clinical outcomes compared to HPV16 positive OPSCC with intact (or mixed) *E2*, we then evaluated if expression of the E6 and E7 oncogenes was associated with clinical outcome. We analyzed all OPSCC samples with available clinical data regardless of *E2* disruption results ranked by level of expression (**Fig 5C and 5D**). We found that tumors derived from the oropharynx which had E7 expression levels below the median cutoff had a higher risk of local-regional recurrence (6C; median = 1.69 2maxΔCT- ΔCT; Mantel-Cox test p = 0.004). Tumors derived from the oropharynx with E6 expression levels below the median cutoff had a higher risk of local-regional recurrence as well (6D; median = 1.23 2maxΔCT- ΔCT; Mantel-Cox test p = 0.006).

## Discussion

HPV16 detection in HNSCC has been associated with improved response to treatment and clinical outcome [5,6]. However, 10–20% of HPV16 positive HNSCC fail to respond to treatment [5]. The HPV16 *E2* gene plays a role in cervical cancer progression and has been shown to regulate expression of the E6 and E7 oncogenes [45,46]. However, the relationship between *E2* disruption and E6/E7 expression has been largely unstudied in HNSCC.

This study evaluates the physical state of the *E2* gene in HPV16 positive HNSCC tumors and assesses the relationship between *E2* disruption and viral oncogene expression as well as clinical outcome. Evaluating 48 HPV16 positive tumors from a cohort of HNSCC patients followed prospectively at a large urban health center, we found that the majority of OPSCC samples had intact or possibly mixed (intact and disrupted) *E2* gene products compared to HPV16 positive non-OPSCC tumors. While seemingly in contrast to what has been reported in cervical cancers, this was in line with recent studies of OPSCC in which episomal or mixed HPV16 (samples containing both episomal and integrated HPV) were detected in the majority of tumors [25,47,48].

The *E2* gene itself is comprised of three domains: a transactivation domain, a DNA-binding domain, and a flexible hinge region which connects the two domains [49]. We independently measured expression of transcripts containing both the transactivation domain and DNA-binding domain of the E2 transcript and found that both measurements showed high levels of transcripts expressed in OPSCCs with an intact *E2* gene compared to samples with a disrupted *E2* gene. This indicates it is likely the full length E2 transcript is present in OPSCCs with an intact gene.

In addition to our findings that the majority of HPV16 positive OPSCC have an intact *E2*, we found that five HNSCC samples harbored *E2* genes with deletions and mutations. Three of these were OPSCCs and two were non-OPSCC. Of the three OPSCC samples, all three involved deletions within the hinge region, which has been reported to harbor deletions in cervical cancer [50–52]. Deletions within the hinge region may hinder nuclear localization of E2 and E2-dependent replication. However, it has also been reported that deletions within the hinge region may not totally abolish the activity of the E2 protein, indicating there may still be a functional E2 protein in these samples [53]. Among the two non-OPSCC tumors, one harbored a deletion in the hinge region and the other a deletion in the transactivation domain. However these *E2* sequences were identified as being non-functional due to mutations within the *E2* gene (Genebank IDs HM162463.1 and HM162476.1) [52].

In addition to viral transcripts, we also assessed the expression of two host transcripts from the CDKN2A locus, p16^INK4A^ and p14^ARF^, that are affected by E6 and E7 deregulation of the p53 and Rb tumor suppressor pathways [54–57]. Expression of these transcripts has been shown to be associated with HPV16 infection in OPSCC [15] and improved clinical outcome in HPV16 negative non-OPSCC [58]. In this study, p16^INK4A^ and p14^ARF^ expression was increased in OPSCC samples with an intact *E2* gene.

Studies in cervical cancer have shown that the presence of a disrupted *E2* gene is associated with poor prognosis [30,31,59] and radioinsensitivity [33]. A similar association between *E2* disruption, local treatment failure and poor survival has also been reported for HPV16 positive OPSCC [60]. We found that OPSCC patients with HPV16 positive tumors harboring a disrupted *E2* had increased local-regional recurrence (p = 0.04) and shortened disease-specific survival (p = 0.03). This was in accordance with a 2001 study of HPV16 positive OPSCC, which found that only those tumors with *E2* disruption had local treatment failure [60]. A separate study found a positive association between E6 and E7 expression levels and improved risk of local-regional recurrence (p = 0.04), but no association with viral integration or detection of extrachromasomal and/or mixed forms of HPV16 [32].

Taken together, these results indicate that detection of disrupted HPV16 *E2* or low levels of E7 expression may be useful in identifying the subset of HPV16 positive OPSCC tumors that are more likely to fail treatment. In this study we evaluated HPV16 positive OPSCC and non-OPSCC tumors. This study has a number of limitations that should be noted. Our stringent criteria for classifying a sample HPV16 positive included multiple HPV16 DNA and RNA tests. This approach was adopted as it was deemed to produce less false positives. However, it is possible HPV16 positive cases were excluded. This approach also reduced our study sample size, which precluded the possibility of conducting multivariable analysis. In addition, the lack of material, particularly for RNA, resulted in smaller sample sizes for correlative analyses between DNA and RNA measures. Our assay for detection of *E2* status did not differentiate between samples with intact only or mixed (intact and disrupted) forms of the gene, nor did we assess for other (e.g., epigenetic) mechanisms that might explain our observed associations between *E2* disruption and E6/E7 expression. We also did not test for the presence of tandemly integrated viral genomes which has been reported in cervical tumors with integrated HPV [61]. Another scenario we did not test for is the existence of viral-human hybrid episomes as described by Nulton et al. [29,61]. These conditions can result in viral genomes with an intact *E2* gene suggesting that the genomes replicate in an E1-E2 dependent manner.

Another recent study by Koneva et al. on HNSCC tumors also found that tumors without HPV integration had improved survival compared to tumors that did have integration or were HPV negative. This study also found that in integration negative samples, immune related genes were the most over expressed group of genes, suggesting that improved survival in this group may be due to increased immunogenicity [62]. An earlier, study by the same group found that there were two distinct subtypes of HPV positive HNSCC tumors based on gene expression. These subtypes were identified as being high expressers of genes related to immune response or keratinocyte differentiation. The subtype associated with immune response was also reported to have less viral integration, higher E2 expression and higher full-length E6 activity compared to the tumors expressing genes for keratinocyte differentiation [63]. Taken together, these two studies indicate that HPV positive HNSCC cases can be stratified and that there appear to be two major carcinogenic pathways. This is similar to results reported in our study which suggest OPSCC cases may be stratified by viral expression or *E2* gene status.

Despite these limitations, our findings indicate that *E2* disruption is uncommon in OPSCC and that the presence of an intact *E2* gene is associated with increased E6 and E7 expression. This is contrary to *in vitro* models of HPV16 negative induced transformation as well as what is reported in many cervical cancer studies, where *E2* disruption is prevalent. However, others have shown that E6 and E7 can be expressed at high levels in the presence of an intact *E2* gene [24,64], which has been attributed to methylation of the *E2* gene binding sites [23,24]. The majority of HPV16 positive non-OP tumors in this study had a disrupted *E2* gene, and *E2* disruption showed no association with viral expression. In contrast, most OPSCC tumors had intact forms of the *E2* gene, which correlated with high viral expression. These tumors also had high expression of host *CDKN2A* (p16^INK4A^ and p14^ARF^) gene expression. We also found that HPV16 positive OPSCCs with disrupted *E2* had poorer cancer prognosis (i.e., shorter disease-specific survival and increased local or regional recurrence), as did OPSCCs with low HPV16 E7 expression. This is in line with previous studies that have shown disruption of the HPV16 *E2* gene to be associated with shorter disease-specific survival in cervical cancer, and with local treatment failure in OPSCC [30,31,60]. With current considerations for dose de-escalation for HPV16 negative associated OPSCC, an assay specific to detection of disruption of the *E2* gene or E6/E7 oncogene expression among HPV16 DNA positive (or p16 protein positive) OPSCC may help identify HPV16 positive OPSCC patients at higher risk of treatment failure.

## Supporting information

S1 Table Primer sequences for HPV16 *E6 and E2 genes*, *and E2* disruption assay. (DOCX)

S2 Table Primer sequences for HPV16 Rt-qPCR assays. (DOCX)

S1 Fig Representative examples of results from PCR for HPV16 *E2* using the full gene primer set, showing disruption, deletion and intact *E2* results in head and neck samples. SiHa and UPCI:SCC090 are used as *E2* disrupted and *E2* intact controls. Column 5, 6, and 7 are results from three different patient samples: column 5 represents a sample which had *E2* disruption, column 6 (red arrow) represents a sample which had a deletion in the *E2* gene and was observed to have a smaller product. The deletion was confirmed by Sanger sequencing. Column 7 represents a sample which had an intact *E2* gene.
(TIF)

S1 File Open data file. Including RNA expression data and viral load data as well as site and *E2* status.
(XLSX)
