# Supplementary material for: Association of an intact E2 gene with higher HPV viral load, higher viral oncogene expression, and improved clinical outcome in HPV16 positive head and neck squamous cell carcinoma
Source: PLoS One. 2018 Feb 16;13(2):e0191581. doi: 10.1371/journal.pone.0191581 (PMC5815588; doi:10.1371/journal.pone.0191581)
Supplement: S1 Table — (DOCX) [file pone.0191581.s001.docx]

**S1 Table: E2 and E6 PCR primers**

| **Target** | **Nucleotide**  **Sequence**  **Location** | **Nucleotide Sequence** | **Amplimer**  **Length**  **(bp)** |
| --- | --- | --- | --- |
| HPV16 E6 | 368  528 | 5’-GAACAGCAATACAACAAACC -3’  5’-GATCTGCAACAAGACATACA -3’ | 161 |
| HPV16 E2  Complete  Gene | 2721  3887 | 5’-TTAAGTTTGCACGAGGACGA -3’  5’-CGCCAGTAATGTTGTGGATG -3’ | 1167 |
| HPV16 E2  Primer set 1 | 2701  3119 | 5’-AGGACGTGGTCCAGATTAAG -3’  5’-TCAAACTGCACTTCCACTGT -3’ | 419 |
| HPV16 E2  Primer set 2 | 3062  3402 | 5’-TAACTGCACCAACAGGATGT -3’  5’-GCCAAGTGCTGCCTAATAAT -3’ | 341 |
| HPV16 E2  Primer set 3 | 3345  3568 | 5’-ATCTGTGTTTAGCAGCAACG -3’  5’-TAAATGCAGTGAGGATTGGA -3’ | 224 |
| HPV16 E2  Primer set 4 | 3542  3785 | 5’-ACAGTGCTCCAATCCTCACT -3’  5’-TCACGTTGCCATTCACTATC -3’ | 244 |
| HPV16 E2  Primer set 5 | 3710  3916 | 5’- GGCATTGGACAGGACATAAT -3’  5’-CAAAAGCACACAAAGCAAAG -3’ | 207 |
