# Supplementary material for: Association of an intact E2 gene with higher HPV viral load, higher viral oncogene expression, and improved clinical outcome in HPV16 positive head and neck squamous cell carcinoma
Source: PLoS One. 2018 Feb 16;13(2):e0191581. doi: 10.1371/journal.pone.0191581 (PMC5815588; doi:10.1371/journal.pone.0191581)
Supplement: S2 Table — (DOCX) [file pone.0191581.s002.docx]

S2 Table: RT-qPCR primers

| Target | Nucleotide  Sequence  Location | Nucleotide Sequence |
| --- | --- | --- |
| HPV16 E2 5’  Forward  Reverse  Probe | 3259  3321 | GTGCAGTTTAAAGATGATGCAGAAAAATATAGT  AGATGTAGGACATAATATTACCTGACCA  CCCGCATGAACTTC |
| HPV16 E2 3’  Forward  Reverse  Probe | 3742  3805 | AGTGCAATTGTTACACTTACATATGATAGTGAA  AGTGCAATTGTTACACTTACATATGATAGTGAA  CAACGTGAACAATTTT |
| HPV16 E6  Forward  Reverse  Probe 1 | 127  184 | ACCCAGAAAGTTACCACAGTTATGC  TGCTTGCAGTACACACATTCTAAT  ACAGAGCTGCAAACAA |
| HPV16 E7  Forward  Reverse  Probe 2 | 653  726 | GCTCAGAGGAGGAGGATGAAATAGA  GAGTCACACTTGCAACAAAAGGTT  TCCGGTTCTGCTTGTCC |
