# Supplementary figures and images for: Association of an intact E2 gene with higher HPV viral load, higher viral oncogene expression, and improved clinical outcome in HPV16 positive head and neck squamous cell carcinoma
Source: PLoS One. 2018 Feb 16;13(2):e0191581. doi: 10.1371/journal.pone.0191581 (PMC5815588; doi:10.1371/journal.pone.0191581)

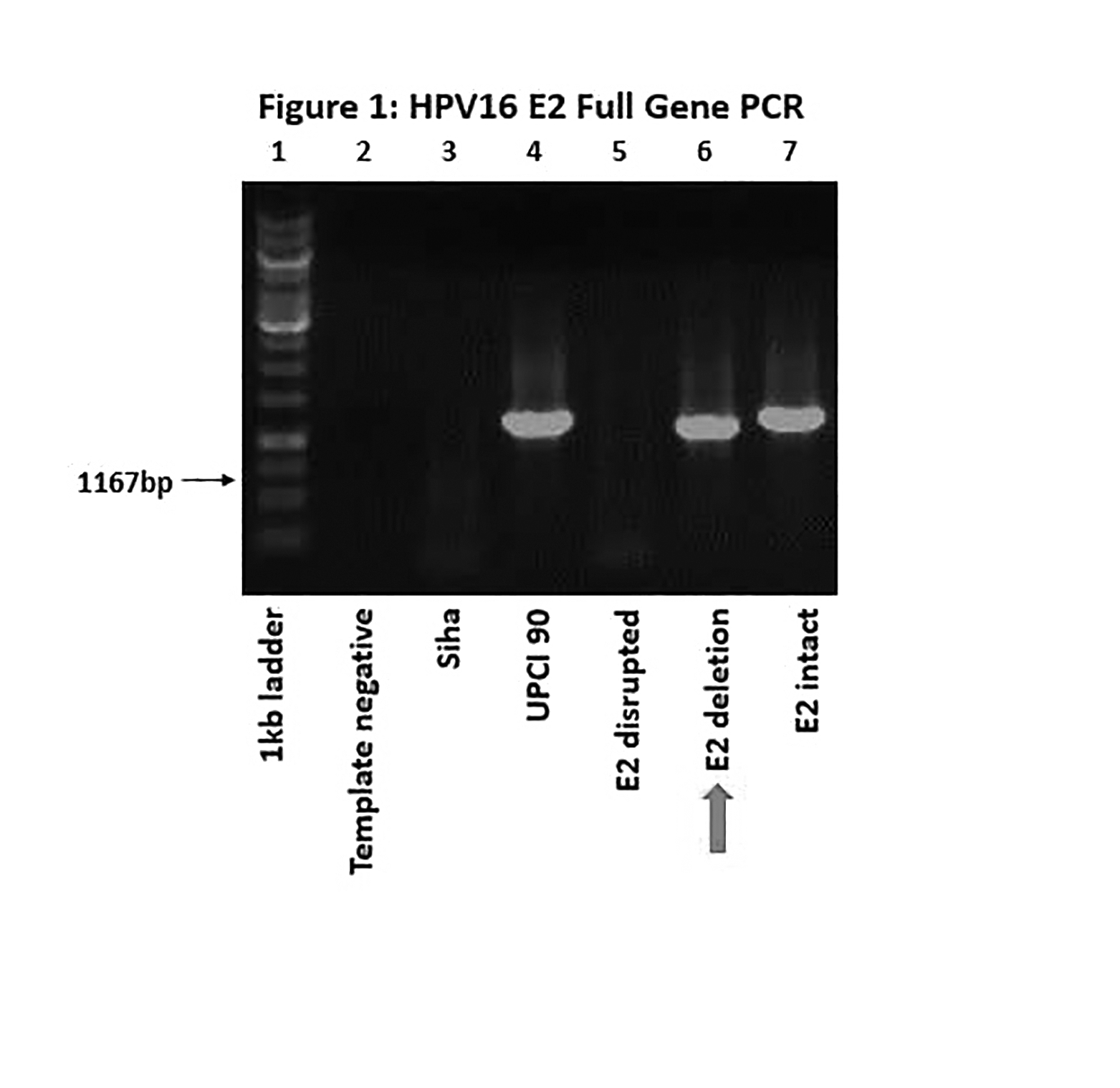

Supplement: S1 Fig — SiHa and UPCI:SCC090 are used as E2 disrupted and E2 intact controls. Column 5, 6, and 7 are results from three different patient samples: column 5 represents a sample which had E2 disruption, column 6 (red arrow) represents a sample which had a deletion in the E2 gene and was observed to have a smaller product. The deletion was confirmed by Sanger sequencing. Column 7 represents a sample which had an intact E2 gene. (TIF) [file pone.0191581.s003.tif]
